# Identification of therapeutic targets and mechanisms of tumorigenesis in non-small cell lung cancer using multiple-microarray analysis

**DOI:** 10.1097/MD.0000000000022815

**Published:** 2020-10-30

**Authors:** Dan Zhao, Hai-Jun Mu, Hai Bing Shi, Hong Xia Bi, Yun Fei Jiang, Guo Hua Liu, Hong Yan Zheng, Bo Liu

**Affiliations:** a School of Medical Technology; b The Third Affiliated Hospital, Qiqihar Medical University, Qiqihar City, PR China.

**Keywords:** multiple-microarray analysis, non-small cell lung cancer, prognostic markers, therapeutic targets

## Abstract

Lung cancer is the most commonly occurring cancer attributed to the leading cause of cancer-related deaths globally. Non-small cell lung cancer (NSCLC) comprises 85% to 90% of lung cancers. The survival rate of patients with advanced stage NSCLC is in months. Moreover, the underlying molecular mechanisms still remain to be understood.

We used 2 sets of microarray data in combination with various bioinformatic approaches to identify the differentially expressed genes (DEGs) in NSCLC patients.

We identified a total of 419 DEGs using the Limma package. Gene set enrichment analysis demonstrated that “Citrate cycle (TCA cycle),” “RNA degradation,” and “Pyrimidine metabolism” pathways were significantly enriched in the NSCLC samples. Gene Ontology annotations of the 419 DEGs primarily comprised “glycosaminoglycan binding,” “cargo receptor activity,” and “organic acid binding.” Kyoto Encyclopedia of Genes and Genomes analysis revealed that DEGs were enriched in pathways related to “Malaria,” “Cell cycle,” and “IL-17 signaling pathway.” Protein protein interaction network analysis showed that the hub genes constituted of CDK1, CDC20, BUB1, BUB1B, TOP2A, CCNA2, KIF20A, CCNB1, KIF2C, and NUSAP1.

Taken together, the identified hub genes and pathways will help understand NSCLC tumorigenesis and develop prognostic markers and therapeutic targets against NSCLC.

## 1 Introduction

Lung cancer is the leading cause for cancer mortality and second most commonly diagnosed cancer globally.^[1]^ However, with the development of efficient therapeutic interventions, lung cancer has become a treatable disease from its status of extremely poor prognosis.^[2]^ Non-small cell lung cancer (NSCLC) comprises 85% to 90% of lung cancers. NSCLC includes adenocarcinoma, squamous cell carcinoma, large cell carcinoma, and other rare subtypes.^[3]^ Therefore, it is important to identify molecular biomarkers and mechanism(s) of pathogenesis associated with NSCLC that will help develop novel therapeutic approaches to improve patient outcome.

The advent of in silico technology has allowed significant progress to be made in understanding the molecular mechanisms and biomarkers involved in a variety of diseases.^[4]^ Gene Expression Omnibus database is a gene expression database created and maintained by NCBI that is widely utilized for identifying key genes and potential mechanisms associated with tumorigenesis and its progression.^[5]^ In this study, we used integrated bioinformatic analysis to determine the biological characteristics of NSCLC. The microarray datasets GSE18842^[6]^ and GSE118370^[7]^ were used to identify the differentially expressed genes (DEGs) in NSCLC. We performed enrichment analysis using Gene Ontology (GO) and Kyoto Encyclopedia of Genes and Genomes (KEGG) pathways to determine potential mechanisms of tumorigenesis in children with NSCLC.^[8]^ Protein-protein interaction (PPI) network analysis using STRING predicted the correlation between the proteins of the DEGs.^[9]^ In summary, we have screened 10 hub genes associated with NSCLC and demonstrated the molecular mechanisms involved in the progression of NSCLC.

## 2 Materials and methods

### 2.1 Microarray analysis

Microarray data were obtained from the Gene Expression Omnibus database (http://www.ncbi.nlm.nih.gov/geo/).^[5]^ The following search terms were used: “NSCLC,” “gene expression,” and “microarray.” We used 2 gene expression datasets (GSE18842 and GSE118370). The platforms used for both datasets were based on the GPL570 [HG-U133_Plus_2] Affymetrix Human Genome U133 Plus 2.0 Array. GSE18842 comprised a total of 91 samples (45 control samples and 46 NSCLC samples) and GSE118370 comprised 12 samples (6 controls and 6 NSCLC samples). This study was approved by the Ethics Committee of The Third Affiliated Hospital, Qiqihar Medical University.

### 2.2 Identification of DEGs

The Limma package was downloaded from Bioconductor for data consolidation (data standardization and log2 conversion) and screening DEGs. A standard of |log2FC| ≥2 and *P*-values < .05 were considered statistically significant. Data was pre-processed by converting probes into gene symbols, data consolidation, and batch normalization by “sva” in the R software (version 3.5.2) and the merged data were devoid of batch effects. After batch normalization, Limma was used to identify DEGs between NSCLC and control samples.^[10]^ Adjusted *P*-values < .05 and |log2FC| ≥2 were considered statistically significant.

### 2.3 Gene set enrichment analysis (GSEA)

GSEA is a method to calculate the statistical significance between a defined set of genes among 2 groups. The reference gene sets in this study was c2.cp.kegg.v6.2. symbols. Gmt using which the normalized enrichment score was calculated. If the *P*- and false discovery rate *q*-values were both <0.05, the gene set was considered to be significantly enriched.^[11]^

### 2.4 GO and KEGG pathway enrichment analysis for DEGs

GO and KEGG pathways were analyzed to identify DEGs by the R software. clusterProfiler was used to identify and visualize the GO terms and KEGG pathways associated with DEGs.^[10]^*P*-value < .05 demonstrated significant enrichment.

### 2.5 PPI network for DEGs

STRING database (STRING11.0; www.string-db.org) was used to predict the DEG PPI network. Interactions with a combined score of >0.9 was the cut-off. PPI networks were generated using the Cytoscape software (version3.7.1). Significant modules and hub genes were confirmed by molecular complex detection (MCODE1.5.1; a cytoscope plug-in).^[12]^ The most significant module identified by MCODE had a score of ≥10.

### 2.6 Hub gene screening

The hub genes were identified using Cytoscape (version 3.7.1). CytoHubba can predict the important nodes and subnetworks in each network employing multiple topological algorithms.^[12]^

## 3 Results

### 3.1 Identification of DEGs in NSCLC

**Figure 1:**
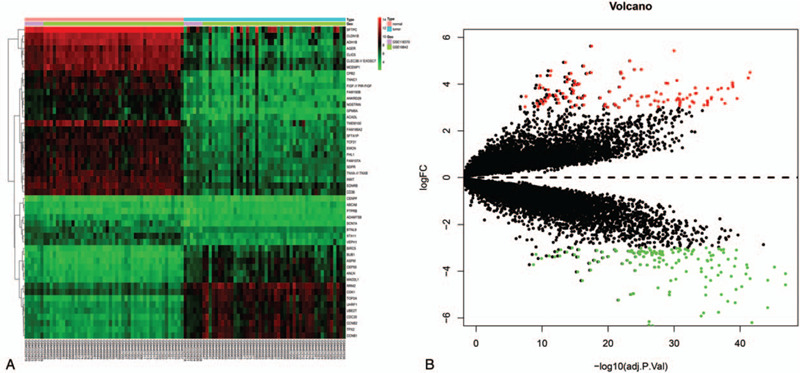
Heatmap of DEGs screened by Limma package. (A) Red areas represent highly expressed genes and green areas represent lowly expressed genes in NSCLC from tumor compared with normal. (B) Volcano plot analysis identifies DEGs. There were including 187 upregulated genes and 232 down regulated genes. Red dots represent upregulated genes and blue dots represent downregulated genes in NSCLC from tumor compared with normal. DEGs = differentially expressed genes, NSCLC = non-small cell lung cancer.

A total of top 50 DEGs were identified as shown in the heatmap (Fig. 1A) that included 187 upregulated and 232 downregulated genes (adjusted *P* < .05, |log2FC| ≥2) as shown in the volcano plot (Fig. 1B).

### 3.2 PPI network analysis

**Figure 2:**
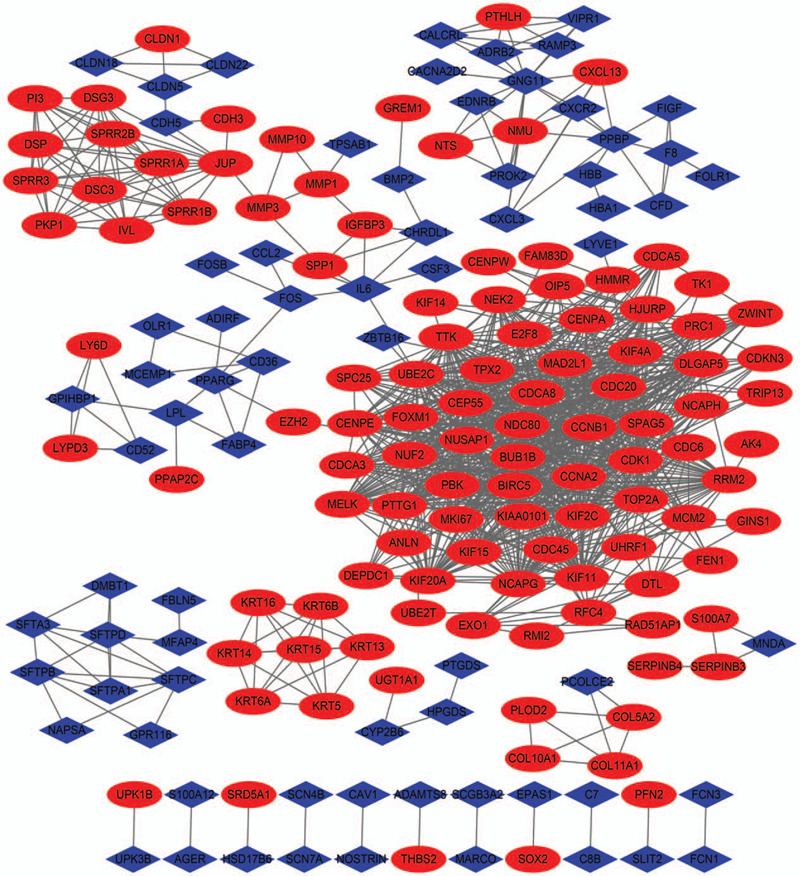
PPI network of DEGs. PPI network of DEGs created by STRING. Cycles represent genes and lines represent PPIs. DEG = differentially expressed gene, PPI = protein-protein interaction.

To identify the most significant DEG clusters, PPI networks, consisting of 190 nodes and 1085 edges, were generated using STRING and Cytoscope (Fig. 2).

### 3.3 Bioinformatic analysis of DEGs

**Figure 3:**
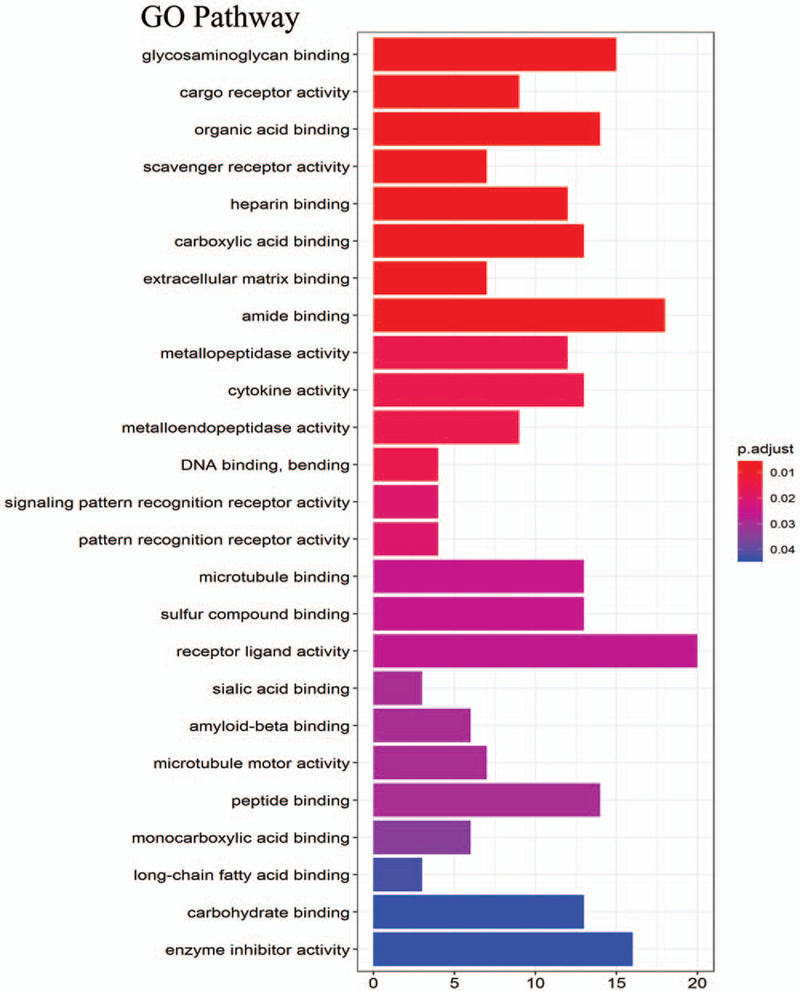
GO enrichment result of DEGs. The *x*-axis represents gene ratio and *y*-axis represents GO terms. The size of cycle represents gene count. Different color of circles represents different adjusted *P*-value. DEG = differentially expressed gene, GO = Gene Ontology.

**Figure 4:**
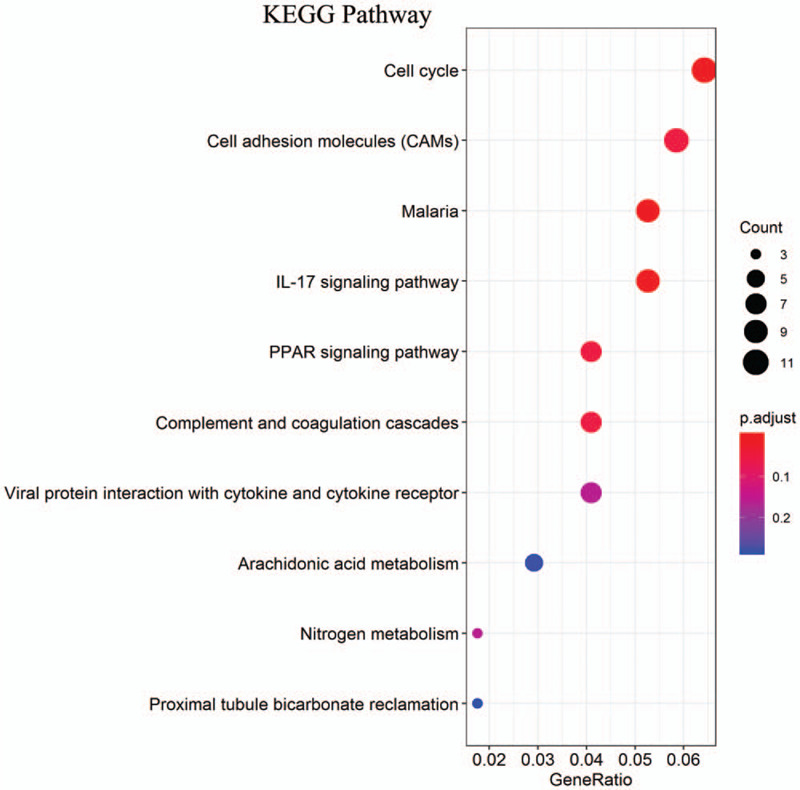
KEGG enrichment result of DEGs. The *x*-axis represents gene ratio and *y*-axis represents KEGG terms. The size of circle represents gene count. Different color of circles represents different adjusted *P*-value. DEG = differentially expressed gene, KEGG = Kyoto Encyclopedia of Genes and Genomes.

The DEGs were analyzed by GO enrichment and KEGG pathway. GO analysis revealed that DEGs were significantly enriched in 25 terms (Fig. 3). The top 3 terms were: “glycosaminoglycan binding,” “cargo receptor activity,” and “organic acid binding.” KEGG analysis revealed that DEGs were significantly enriched in 10 terms (Fig. 4). The top 3 terms were: “Malaria,” “Cell cycle,” and “IL-17 signaling pathway.”

### 3.4 GSEA

**Figure 5:**
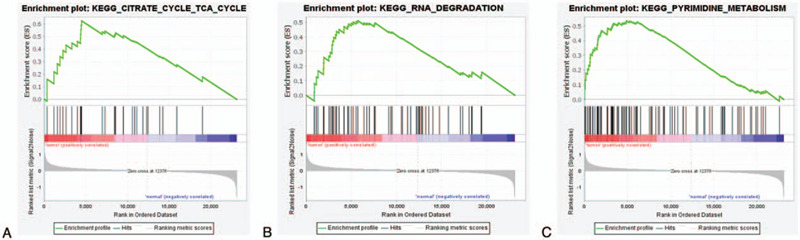
GSEA plot showing most enriched gene sets in NSCLC from tumor compared with normal. (A) The most significant enriched gene set positively correlated with NSCLC was Citrate cycle (TCA cycle) (NES = 1.90, *P*-value < .05, FDR *q*-value < 0.05); (B) The second significant enriched gene set positively correlated with NSCLC was RNA degradation (NES = 1.80, *P*-value < .05, FDR *q*-value < 0.05); (C) The third significant enriched gene set positively correlated with NSCLC was Pyrimidine metabolism (NES = 1.76, *P*-value < .05, FDR *q*-value < 0.05). FDR = false discovery rate, GSEA = gene set enrichment analysis, NES = normalized enrichment score.

GSEA was performed to explore the underlying biological functions involved in NSCLC; “Citrate cycle (TCA cycle),” “RNA degradation,” and “Pyrimidine metabolism” were the significantly enriched gene sets that correlated with NSCLC (*P* < .05; Fig. 5).

### 3.5 Hub gene analysis

**Figure 6:**
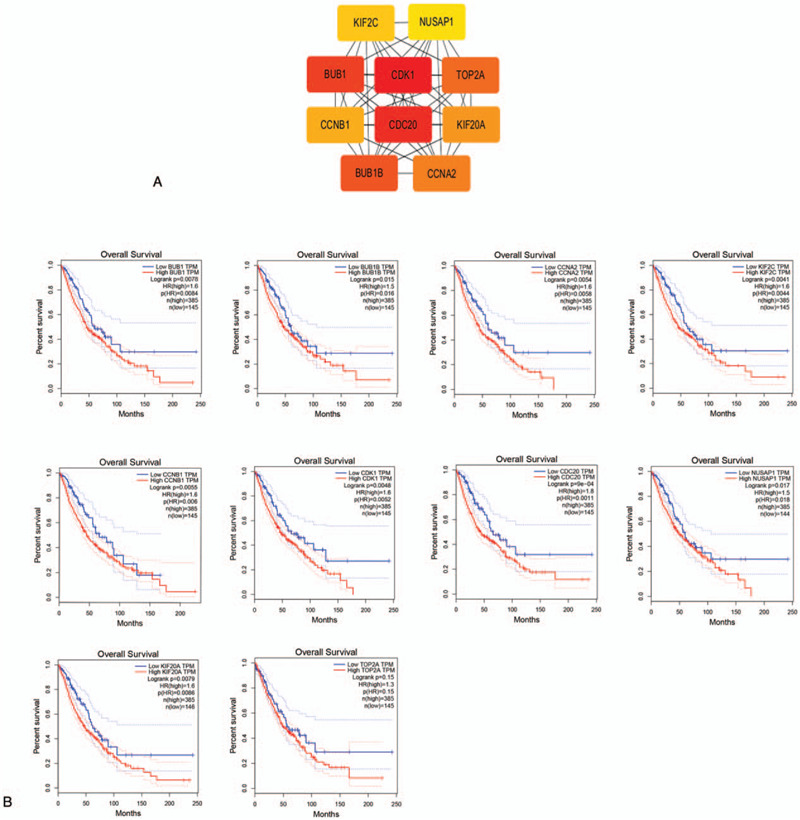
The most significant module of DEGs: (A) The PPI network of DEGs was performed using Cytoscape and the most significant module was obtained from PPI network with 10 nodes. (B) Overall survival analysis for 10 hub genes in lung cancer from the GEPIA tool. DEG = differentially expressed gene, PPI = protein protein interaction.

We downloaded the STRING-generated PPI network of 419 DEGs and then used Cytoscape to construct the whole network. The most significant module of the whole network comprised 10 nodes, that is, hub genes (Fig. 6A). To evaluate the prognostic value of hub genes in lung cancer patients, we performed log-rank survival curve analysis using data from TCGA RNA sequencing datasets (GEPIA tool). The overall survival of patients with higher expression of the lung cancer hub genes was shorter than patients with lower expression of the hub genes (Fig. 6B).

## 4 Discussion

The last 2 decades have seen significant advancements in the treatment of NSCLC, thereby improving our understanding of the biology of NSCLC and mechanisms of tumor progression, early detection, and multimodal care.^[13]^ The use of small molecule tyrosine kinase inhibitors and immunotherapy has led to unprecedented survival benefits in patients. However, success of treatment and survival rates for NSCLC patients still remain low (especially for metastasized tumors).^[14]^ Therefore, there is a need for the development of novel drugs and combination therapies to allow benefits to a wider patient population and improve outcomes in NSCLC patients. We have focused on bioinformatic analysis to identify DEGs between NSCLC and normal individuals.

In this study, we analyzed data from 2 microarray datasets that identified a total of 419 DEGs (187 upregulated and 232 downregulated genes). Among these, 10 candidates comprised the hub genes. GO enrichment of DEGs revealed that they were mostly enriched in glycosaminoglycan binding,^[15]^ cargo receptor activity,^[16]^ and organic acid binding^[17]^; KEGG enrichment showed predominant functional enrichment in malaria,^[18]^ cell cycle,^[19]^ and IL-17 signaling pathway.^[20]^ GSEA showed that NSCLC was associated primarily with the citrate cycle (TCA cycle),^[21]^ RNA degradation,^[22]^ and Pyrimidine metabolism pathway.^[23]^ Previous studies have shown that these GO, KEGG, and GSEA pathways play important roles in the development and progression of tumors.

All 10 hub genes may be key biomarkers in NSCLC tumorigenesis The genes were CDK1, CDC20, BUB1, BUB1B, TOP2A, CCNA2, KIF20A, CCNB1, KIF2C, and NUSAP1. CCNB1 affects NF-kB signaling that is associated with angiogenesis.^[24]^ We used PubMed and GeneCards to understand the biological function of TOP2A. TOP2A has been studied the most among the hub genes and it affects multiple cancer types. Raghavan et al (2012) demonstrated that CDK1 enhances the radioresistance of NSCLC cells.^[25]^ Overexpression of CDC20 correlates with the poor prognosis of primary NSCLC patients.^[26]^ Bub1 inhibition and resulting activation of DNA damage signaling regulates the localization of apoptin.^[27]^ Lee et al (2017) have shown that BUB1B was associated with poor survival rates, possibly because such patients tended to have more invasive disease.^[28]^ TOP2A induces drug resistance via Wnt signaling in NSCLC.^[29]^ Biagioni et al (2012) showed that CCNA2 affects cell cycle regulation.^[30]^ KIF20A overexpression confers a malignant phenotype to ovarian tumors by promoting proliferation and inhibiting apoptosis.^[31]^ Lu et al (2014) revealed that KIF2C promotes the rapid restructuring of the microtubule cytoskeleton.^[32]^ NUSAP1 is involved in the development, progression, and metastasis of multiple cancers.^[33]^ These proteins play an important role in various tumors, especially NSCLC, and may help identify specific and sensitive diagnostic markers and therapeutic targets.

## 5 Conclusion

This study identified a host of DEGs and hub genes in NSCLC patients that may act as candidates for biomarkers and therapeutic targets, thereby enabling a better understanding of the molecular mechanism(s) in NSCLC. However, the study has some limitations, such as a relatively small sample size and lack of knowledge on the correlation between these genes and clinical information. Thus, further experiments are needed to verify the candidates in this study and determine their biological roles in NSCLC.

## Author contributions

Hai-Jun Mu was responsible for the methodology. Dan Zhao, Yun Fei Jiang, and Guo Hua Liu were involved in the investigation. Dan Zhao and Hai-Jun Mu wrote the manuscript. Hai Bing Shi, Bo Liu, Hong Yan Zheng, and Hong Xia Bi were involved in conceptualization and review & editing of manuscript. All the authors have read and approved the final manuscript.
